# *Piper aduncum* Essential Oil Rich in Dillapiole: Development of Hydrogel-Thickened Nanoemulsion and Nanostructured Lipid Carrier Intended for Skin Delivery

**DOI:** 10.3390/pharmaceutics14112525

**Published:** 2022-11-19

**Authors:** Simone Braga Carneiro, Tainá Kreutz, Renata Pereira Limberger, Helder Ferreira Teixeira, Valdir Florêncio da Veiga Júnior, Letícia Scherer Koester

**Affiliations:** 1 Programa de Pós-Graduação em Inovação Farmacêutica, Faculdade de Ciências Farmacêuticas, Universidade Federal do Amazonas, Av. Gal. Rodrigo Octávio, Coroado I, 1200, Manaus 69067-005, Brazil; 2 Programa de Pós-Graduação em Ciências Farmacêuticas, Faculdade de Farmácia, Universidade Federal do Rio Grande do Sul, Av. Ipiranga, Santana, 2752, Porto Alegre 90610-000, Brazil; 3 Programa de Pós-Graduação em Química, Instituto Militar de Engenharia, Praça General Tibúrcio, Urca, 80, Rio de Janeiro 22290-270, Brazil

**Keywords:** essential oil, *Piper aduncum*, dillapiole, nanotechnology, skin permeation, anti-inflammatory

## Abstract

The essential oil extracted from the leaves of *Piper aduncum*, an aromatic plant from the Amazon region, is rich in dillapiole and presents anti-inflammatory activity. In this study, nanoemulsions (NE) and nanostructured lipid carriers (NLC), which are biocompatible nanostructured systems of a lipid nature, were prepared by high-pressure homogenization for the yet unexplored skin delivery of dillapiole. The addition of hydroxyethylcellulose produced hydrogel-thickened NE or NLC in view to improving the viscosity and skin adherence of the nanoformulations. Formulations were characterized with respect to dillapiole content, droplet size, polydispersity index, zeta potential, morphology, rheological behavior, bioadhesion, skin permeation profile, and in vitro irritancy (HET-CAM). The formulations developed presented spherical, homogeneous nanometric particle size (around 130 nm), narrow polydispersity index (<0.3), and negative zeta potential (around −40 mV). Dillapiole content was slightly lower in NLC compared to NE since the production process involves heating. The hydrogels containing nanocarriers showed pseudoplastic behavior with bioadhesive characteristics. The developed formulations exhibited a controlled release profile, dillapiole delivery up to the dermis, the layer of interest for anti-inflammatory potential, and low irritant potential in the chorioallantoic membrane (HET-CAM). Both hydrogels-thickened NE and NLC seemed to be promising formulations for skin delivery of *Piper aduncum* essential oil.

## 1. Introduction

**Figure 1:**
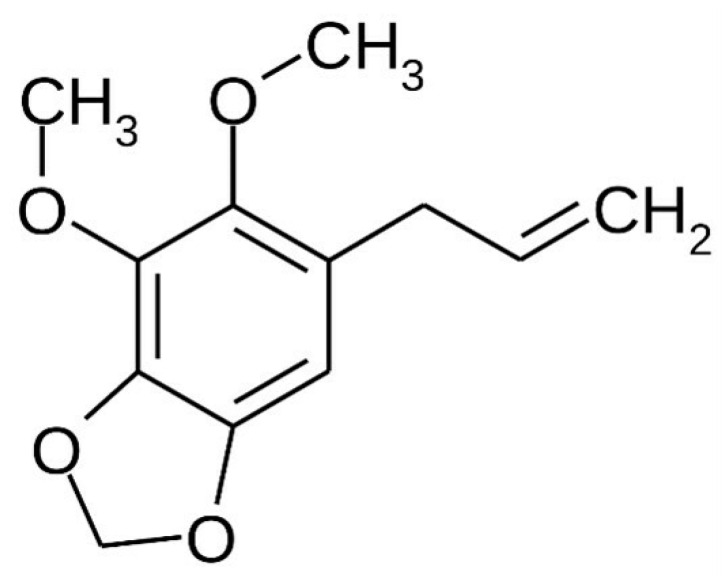
Chemical structure of dillapiole.

The Piperaceae family has its botanical species distributed mainly in the Atlantic Forest and Amazon region and are great producers of essential oils extracted mainly from the leaves [1,2,3,4,5]. The phenylpropanoid dillapiole (Figure 1) is one of the most important substances in the genus Piper, is found in abundance in the species *Piper aduncum* L. in concentrations ranging from 30% to 97% [1,2,3,5], and has been successfully tested as fungicide [6,7,8], insecticide [9], bactericidal [10,11] and anti-inflammatory [12]. As far as we are concerned, there are no studies that address the development of formulations for skin delivery of *P. aduncum* essential oil or dillapiole, nor with the purpose of treating inflammatory processes.

The incorporation of volatile oils in nanostructured systems has gained interest in the last few years since they have been able to increase their bioefficacy. This is related to its capacity to be absorbed by cells and permeate through membranes and biological barriers due to the small particle size and high surface area [13,14,15,16,17]. Moreover, the encapsulation of volatile compounds may confer benefits such as higher stabilization (reduced volatilization) and lower sensitization [14,15,18,19,20,21,22]. Among nanostructured systems, nanoemulsions (NE) are more often requested for volatile oils incorporation than other systems [14,15]. This is probably related to its easy preparation/scaling up and use of biocompatible excipients. Moreover, the preparation of nanoemulsions by high-pressure homogenization may not require heat or solvent evaporation (as in nanocapsules), thus preventing oil volatilization during preparation [22,23,24]. Solid lipid nanoparticles are another kind of lipid nanostructure where part of the liquid lipid is replaced by a solid one [25,26,27]. When both a liquid lipid and a solid lipid are present, the system is called a nanostructured lipid carrier (NLC) [27,28]. The solid structure around the volatile oil in an NLC may control drug release and form an occlusive barrier that promotes drug permeation, among other advantages [27,28,29]. Care must be taken not to use a solid lipid that requires high temperatures for melting; otherwise, volatilization may occur [15,26,27,28]. Within this context, cupuaçu butter, a natural product of vegetal origin from the Amazon, was tested due to its low melting point (approximately 30 °C) [30,31]. To overcome the low viscosity of these nanoformulations and improve their adherence to the skin, NE and NLC are often thickened by the addition of polymers to form hydrogels that provide a pleasant sensory aspect for application on the skin [32,33,34,35].

In this context, the objectives of this study were to evaluate the feasibility of preparing nanoemulsions (NE) and nanostructured lipid carriers (NLC) and respective hydrogels (HNE and HNLC) containing the essential oil of *P. aduncum*, as well as to assess its safety profile by HET-CAM and permeation profile by in vitro permeation assay using Franz cells in view to develop an anti-inflammatory formulation for skin delivery.

## 2. Materials and Methods

### 2.1. Chemicals and Reagents

Span 80^®^, Tween 20^®^ were purchased from Sigma-Aldrich (São Paulo, Brazil). Tween 80^®^ was purchased from Synth (Diadema, Brazil). Cupuaçu butter, olive oil, and caprylic/capric triglyceride (MCT-medium chain triglycerides) were purchased from Mapric (São Paulo, Brazil). Hydroxyethylcellulose (1.0 × 10^6^ Da, viscosity of 1500–2500 cP) was purchased from Pharma Nostra (São Paulo, Brazil). Olive oil was purchased from Alpha Química (Porto Alegre, Brazil). Ultrapure water was obtained from a MilliQ^®^ apparatus (Millipore, Billerica, MA, USA). HCl, NaOH, NaCl, Na_2_HPO_4_, KH_2_PO_4_, sodium lauryl sulfate, hexane, and methanol were all from analytical grade.

### 2.2. Essential Oil from P. aduncum Leaves

Leaves of *P. aduncum* L. were collected at Universidade Federal do Amazonas, located in Manaus, Amazon State, Brazil (3°06′04.0″ S 59°58′40.0″ W), on a sunny afternoon in November 2016. A voucher specimen was deposited at Instituto Nacional de Pesquisas da Amazônia (INPA) Herbarium, under the identification number 205937. Previously, the research with genetic material from Brazilian biodiversity was registered in the Sistema Nacional de Gestão do Patrimônio Genético e do Conhecimento Tradicional Associado (SisGen n° AD9ABC5).

The leaves of *P. aduncum* were dried at room temperature, milled, weighed (totaling 777.32 g), and submitted to extraction under semi-industrial extractors, using 300 g of dried leaves each extraction. Thus, the essential oil of *P. aduncum* (EOPA) with a light yellow coloration and woody smell was obtained as the raw material. Oil composition (with the major compounds: dillapiole (80.05%) and β-caryophyllene (10.65%)) was characterized by gas chromatograph coupled with mass spectrometry QP2010MS (GC-MS, Shimadzu, Kyoto, Japan) and can be found in Figure S1. A dilution of EOPA with hexane was used (1 mg/mL, *w/v*). A TR-5 fused silica capillary column with dimensions of 30 m × 0.25 mm × 0.25 µm was used for chromatographic separation. The carrier gas was ultrapure helium with a flow rate of 1.0 mL/min. The injector temperature was set at 250 °C and splitless mode was used. The oven temperature was programmed as follows: 90 °C to 160 °C (2.5 °C/min) and 160 °C to 290 °C (10 °C/min). The samples were ionized by electron impact with 70 eV energy. The signals were recorded and processed by Thermo Fisher Scientific Software. The linear retention indices (LRI) were obtained for all volatile constituents using a homologous series of n-alkanes. Identification of compounds were made by comparison of mass spectra and LRI with those stored in NIST Mass Spectral Search Program 2.0 Database and in the literature [36].

### 2.3. Chromatographic and Analytical Conditions

Dillapiole (DIL) content from EOPA in formulations and biological matrices was determined by a Shimadzu GC 2010 (Kyoto, Japan) gas chromatograph (GC), with manual sampler, equipped with a flame ionization detector (FID). The injection was made in the splitless mode. The GC system was equipped with DB-5 fused silica capillary column (30 m × 0.25 mm × 0.25 µm). The carrier gas was ultrapure nitrogen (1.0 mL/min) and the linear velocity was 32 cm/s. The oven temperature was programmed as follows: 60 °C for 1 min, from 60 °C to 250 °C at 50 °C/min, from 250 °C to 265 °C at 5 °C/min and from 265 °C to 280 °C at 50 °C/min, staying at 280 °C for 2 min, finalizing the chromatographic run at 10.10 min. Injector and detector temperatures were set at 250 °C and 300 °C, respectively. The signal was recorded and processed with LabSolutions GC-Solution software. The analysis of DIL was made by headspace extraction procedure (HS), under optimized conditions and with a 1 mL syringe. Extraction was made with 10 mL vial sealed with septa and screw cap, under 100 °C and agitation, during 10 min, with NaCl addition of 30% *w/v* and 100 µL of the reference or sample solution. After extraction, 1 mL of the sample vaporized inside the vial was aspirated, inserted into the injection port of the GC, and analyzed. The method was previously validated under analytical and bioanalytical criteria in order to assess DIL content in solutions, formulations, and skin samples with respect to selectivity, linearity, limit of detection (LOD), limit of quantification (LOQ), precision, accuracy, recovery, and carry-over.

### 2.4. Preparation of Nanoemulsion and Nanostructured Lipid Carrier

To assess the impact on the volatilization and in vitro application of different lipid nanosystems containing EOPA, a nanoemulsion, and a nanostructured lipid carrier were developed. The components of both lipid nanosystems are highlighted in Table 1.

In order to prepare the nanoemulsion (NE), aqueous phase containing Tween 20^®^ and ultrapure water and oily phase containing the EOPA, MCT and Span 80^®^ were weighed and mixed separately for 5 min. Then, the aqueous phase was poured in the oily phase, under magnetic stirring at room temperature, to form a coarse emulsion. So, this coarse emulsion was submitted to Ultra-Turrax^®^ T8 (IKA, Staufen, Germany) for 1 min at 13,500 rpm and further to high-pressure homogenization (Emulsiflex-C^®^, Avestin, Ottawa, ON, Canada) for 6 cycles at 750 bars to reduce droplet size. A blank nanoemulsion (B-NE) was also prepared at the same conditions, except it did not contain EOPA.

**Figure 2:**
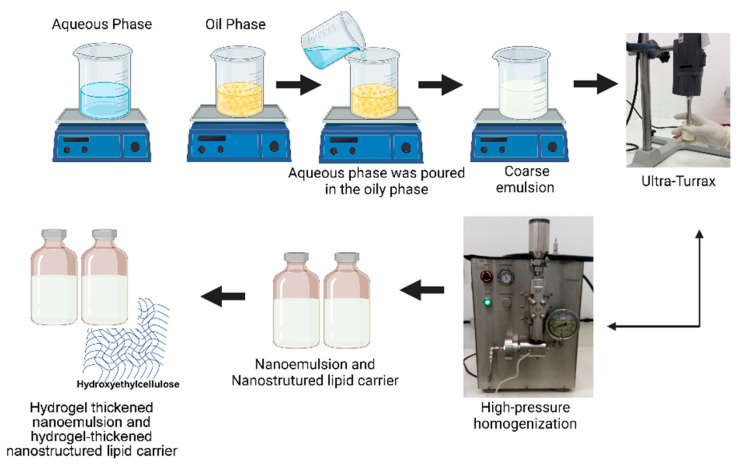
Scheme of preparation of nanoemulsion, nanostructured lipid carrier, and respective hydrogels.

Nanostrutured lipid carrier (NLC) were prepared in the same way, replacing part of MCT by a solid lipid (Cupuaçu butter), which required heating during preparation (45 °C ± 5 °C). Moreover, a blank nanostructured carrier lipid (B-NLC) was also prepared, substituting EOPA for MCT, maintaining the oily core at the same proportion. Figure 2 summarizes the preparation of the nanosystems.

All formulations were prepared in triplicate, stored under refrigeration (4 °C ± 2 °C), and protected from light.

EOPA: essential oil of *P. aduncum*; MCT: medium chain triglyceride; q.s.: quantum satis; B-NE: blank nanoemulsion; B-NLC: blank nanostructured lipid carrier; NE: nanoemulsion containing EOPA; NLC: nanostructured lipid carrier containing EOPA; HB: blank hydroxyethylcellulose hydrogel; HB-NE: blank hydrogel-thickened nanoemulsion; HB-NLC: blank hydrogel-thickened nanostructured lipid carrier; HNE: hydrogel-thickened nanoemulsion containing EOPA; HNLC: hydrogel-thickened nanostructured lipid carrier containing EOPA.

### 2.5. Preparation of Hydroxyethylcellulose Hydrogels Containing Lipid-Based Nanosystems

Hydroxyethylcellulose hydrogels were prepared by adding 1% *w/w* of the polymer directly to the formulation (NE, NLC, B-NE, B-NLC) and left under magnetic stirring for 1 h. The hydrogel formed was left to swell overnight at 4 ± 2 °C. Further, the pH was adjusted close to skin pH (around 5) with hydrochloric acid 1 M or NaOH 0.1 M.

All hydrogels were prepared in triplicate, stored under refrigeration (4 °C ± 2 °C) and protected from light. Composition of thickened formulations are presented in Table 1.

### 2.6. Characterization of Lipid Nanosystems and Derived Hydrogels

After preparation, lipid nanosystems and derived hydrogels were characterized by organoleptic aspects, such as color, odor and appearance, DIL content relative to the initial value incorporated (%), droplet size, polydispersity index (PDI), zeta potential, and morphology.

#### 2.6.1. Evaluation of DIL Content in Lipid Nanosystems and Derived Hydrogels

The quantification of the main marker of EOPA, dillapiole, was determined by HS-GC-FID. Briefly, 1 g of the lipid nanosystem or derived hydrogel was weighed in a 5 mL calibrated volumetric flask and filled with methanol to obtain a stock solution with a theoretical concentration of 10,000 µg/mL from the essential oil. Further, a dilution in water was performed in a 10 mL calibrated flask, obtaining a DIL concentration of 80.07 µg/mL. Then, 100 µL of this reference solution was added to a 10 mL vial, and DIL content was determined according to the methodology described in Section 2.3. Results are expressed as mean ± standard deviation (SD) of percentage content relative to the initial value incorporated (%).

#### 2.6.2. Droplet Size, Polydispersity Index, and Zeta Potential

Droplet size and polydispersity index were measured in triplicate by dynamic light scattering (DLS) at 25 ± 0.5 °C after dilution of 10 µL of the nanoemulsion or nanostructured lipid carrier in 10 mL of purified water filtered with polyvinylidene fluoride (PVDF) 0.45 µm pore membrane filter (1:1000) (Zetasizer Nanoseries ZN90, Malvern Instruments, Worcestershire, UK). The zeta potential was measured in triplicate with the same equipment by electrophoretic light scattering at 25 ± 0.5 °C after dilution of 10 µL of the nanoemulsion or nanostructured lipid carrier in 10 mL of NaCl (1 mM) filtered with PVDF 0.45 µm-pore membrane filter (1:1000). Measurements are shown as mean ± SD.

#### 2.6.3. Morphology of Formulations

The size and morphology of nanoemulsion, nanostructured lipid carrier, and hydrogels containing EOPA were examined by transmission electron microscopy (TEM) using a JEM-1200 EXII microscope (Jeol, Tokyo, Japan), operating at an accelerating voltage of 80 kV. For the analyses, the samples were diluted with ultrapure water (1:25, *v/v*), distributed on formvar-coated copper grids (400 mesh), and stained with uranyl acetate aqueous solution (2%).

### 2.7. Rheological Behavior of Hydrogels

The rheological profile of hydrogels (HB, HB-NE, HB-NLC, HNE, and HNLC) was determined at 25 ± 0.5 °C by a Brookfield DV-II+ Rotational Viscometer (Brookfield Engineering Laboratories, Middleboro, MA, USA), with spindle SC-18. Rheological behavior was carried out with 10 g of samples, in triplicate, with rotation speeds increasing progressively from 6 to 50 rpm and gradually decreasing to 6 rpm, respecting the limits of values of torque (above 10% and less than 100%). The rheograms are presented as shear stress (D/cm^2^) vs. shear rate (1/s) and viscosity (centipoise, cP) vs. shear rate (1/s), and results are expressed as mean ± SD. Further, the rheological flow behavior was determined after fitting the results in different mathematical flow models (Bingham, Ostwald, Casson, and Herschel-Bulkley).

### 2.8. Bioadhesion Measurements

**Figure 3:**
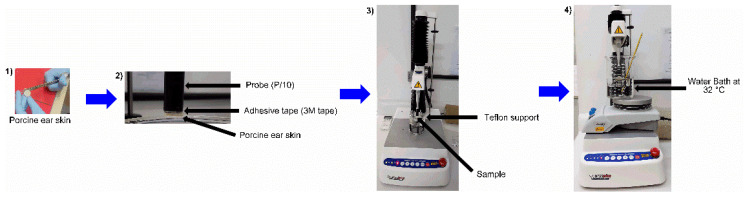
Photographs of the bioadhesion assay: (1) Porcine ear is cut; (2) Porcine ear is fixed in the probe with adhesive tape; (3) Equipment is mounted and sample is placed; (4) The Teflon Support is immersed in a water bath and the test is performed.

Bioadhesive properties of formulations (NE, NLC, HNE, and HNLC) were evaluated by a tensile stress tester (TA.XTplus Texture Analyzer, Stable Microsystem, Godalming, UK). The experiment was performed with porcine ear skin obtained as described in Section 2.10 and left hydrating for 30 min in phosphate buffer 7.4 before the experiment. The porcine skin was fixed in a probe (P/10) with the aid of an adhesive tape (3 M tape) and heated until 32 ± 2 °C before mounting the equipment. In the Teflon support immersed in a water bath at 32 ± 2 °C, positioned below the probe, 400 µL of the formulation was added (NE, NLC, HNE, or HNLC). The probe was lowered at a constant speed of 1 mm/s until it reached the porcine skin. A contact force of 9.8 mN was applied against the skin for 1 min. Further, the probe was lifted with a trigger force of 1 mN and a constant speed of 0.5 mm/s until the complete separation of formulation and porcine skin. The work of adhesion (nM·mm), a direct measurement of bioadhesion [37], and the maximum force of detachment (mN) were determined and applied to compare the bioadhesive properties of the formulations. Figure 3 discloses how the assay is performed. Results are expressed as mean ± SD (*n* = 6).

### 2.9. Release Studies

A released study of DIL from EOPA, NE, NLC, HNE, and HNLC was performed through a synthetic cellulose ester membrane (50 nm pore diameter, Millipore^®^, Burlington, VT, USA) in a manual system of Franz-type diffusion cell with 1.77 cm^2^ of diffusion area (VhTex^®^, Florianópolis, Brazil). Prior, the synthetic membranes were hydrated for 30 min with phosphate-buffered saline (PBS) pH 7.4. The receptor compartment consisted of 12 mL of receptor fluid (a mixture of PBS pH 7.4 and 0.3% *v/v* of Tween 80^®^) and was kept under agitation (500 RPM) and controlled temperature (32 ± 1.0 °C). To the donor compartment, 15 µL of EOPA (corresponding to 5% of EOPA in the oily core of formulations) or 300 µL of NE, NLC, HNE, and HNLC were placed. The experiment, which aimed to estimate the content of DIL released from EOPA and formulations, lasted 12 h and, from time to time, 0.5 mL of the receptor fluid was withdrawn for analysis, and the same volume of fresh fluid was replaced to maintain the sink condition. At the end of the experiment, aliquots of receptor media were analyzed by HS-GC-FID, as described in Section 2.3. Results are expressed as a percentage of the release of DIL (mean ± SD, *n* = 3).

### 2.10. In Vitro Porcine Skin Permeation/Retention Study

Porcine ears were obtained from Ouro do Sul (Cooperativa dos Suinocultores do Caí Superior Ltd., Harmonia, Brazil). The skin was excised from the outside of the ear with the aid of a scalpel. Porcine ear skin was cut in circular slices with a thickness between 0.9 and 1.1 mm (thickness gauge N. 7301, Mitutoyo Corporation, Sakado, Japan). The skin was cleaned with water, and the hypodermis layer and superficial hair were removed with scissors and a scalpel. The skin samples were stored in a freezer (−20 ± 2.0 °C) until use.

The receptor fluid for the permeation/retention experiments consisted of 12 mL of phosphate-buffered saline (PBS) pH 7.4 containing 0.3% *v/v* of Tween 80^®^, which was kept under agitation (500 RPM) and controlled temperature (32 ± 1.0 °C).

Skin permeation/retention study was performed using porcine ear skin (*n* = 5) in a manual system of Franz-type vertical diffusion device with 1.77 cm^2^ of diffusion area (VhTex^®^, Brazil). EOPA or formulations were added directly to the skin in the donor compartment (15 or 300 μL, respectively), which was previously thawed and hydrated in phosphate-buffered saline (PBS) pH 7.4. After 8 h, the skin was removed from the apparatus and the excess of essential oil or formulation was washed from its surface using purified water. The excess skin was cut with the aid of scissors, retaining the portion in contact with the formulation. In addition, the tape-stripping technique was employed at the viable skin to separate the stratum corneum from the other layers (3 M Scotch tape n° 750) [38]. Epidermis and dermis were separated after scraping with a scalpel. Tapes (containing stratum corneum), epidermis, dermis, and fluid aliquots were frozen (−20 °C) for further analysis by HS-GC-FID. All samples were placed directly into the vials without addition of solvents, maintaining the aqueous content of 100 µL.

The analysis of DIL was determined by headspace extraction procedure (HS), under optimized conditions and with a 1 mL syringe. Extraction was made with a 10 mL vial sealed with septa and screw cap, under 100 °C and agitation, for 10 min, with the addition of 100 µL NaCl 30% solution (*w/v*). After extraction, 1 mL of the sample vaporized inside the vial was aspirated, inserted into the injection port of the GC, and analyzed as described in Section 2.3. Results are expressed as a percentage of the release of DIL (mean ± SD, *n* = 5).

### 2.11. HET-CAM Toxicity Test

The Hen’s Egg Chorioallantoic Membrane Test (HET-CAM) was performed to identify the irritant potential of the developed formulations [39,40]. Fertilized hen’s eggs were donated from Mercoaves (Bom Princípio, Brazil) and were incubated at 37 ± 1 °C and 60–70% humidity for 10 days in an automatic incubator (Chocar Chocadeiras, Conceição do Coité, Brazil). On the 10th day, to ensure fertility, the eggs were candled prior to the experiment. Further, the eggs had the shell removed at the air cell and the inner membrane removed, exposing the chorioallantoic membrane (CAM). Then, 300 µL of test substance or formulation was placed on the CAM, and vascular reactions were observed during 300 s (*n* = 5). NaCl solution 0.9% *w/v* was applied as a negative control, while sodium lauryl sulfate 1% *w/v* and NaOH 0.1 M were used as positive controls. Olive oil was employed as a non-irritant diluent for EOPA and was also adopted as negative control [41]. Due to the opacity of B-NE, B-NLC, NE, NLC, HB-NE, HB-NLC, HNE, and HNLC, formulations were rinsed 20 s after application with NaCl solution 0.9 *w/v,* and vascular alterations were also evaluated. Endpoints of hemorrhage, vasoconstriction, and coagulation of each group were monitored in order to calculate the irritation score (IS), as following Equation (1):

IS = [5 × (301 − hemorrhage time)/300] + [7 × (301 − vasoconstriction time)/300] + [9 × (301 − coagulation time)/300](1)

The results were expressed as the mean of IS and relative standard deviation in percentage (RSD%) and were classified by the mean as non-irritant [0.0–0.9], slightly irritant [1.0–4.9], moderately irritant [5.0–8.9], and extremely irritant [9.0–21.0].

### 2.12. Statistical Analysis

Statistical analyses were performed in GraphPad Prism^®^ Software v.06 (GraphPad Software, San Diego, CA, USA) by one-way analysis of variance (ANOVA) followed by Tukey’s post hoc test (significance level *p* ≤ 0.05).

## 3. Results and Discussion

### 3.1. Characterization of Nanoemulsions and Nanostructured Lipid Carriers Containing EOPA

**Figure 4:**
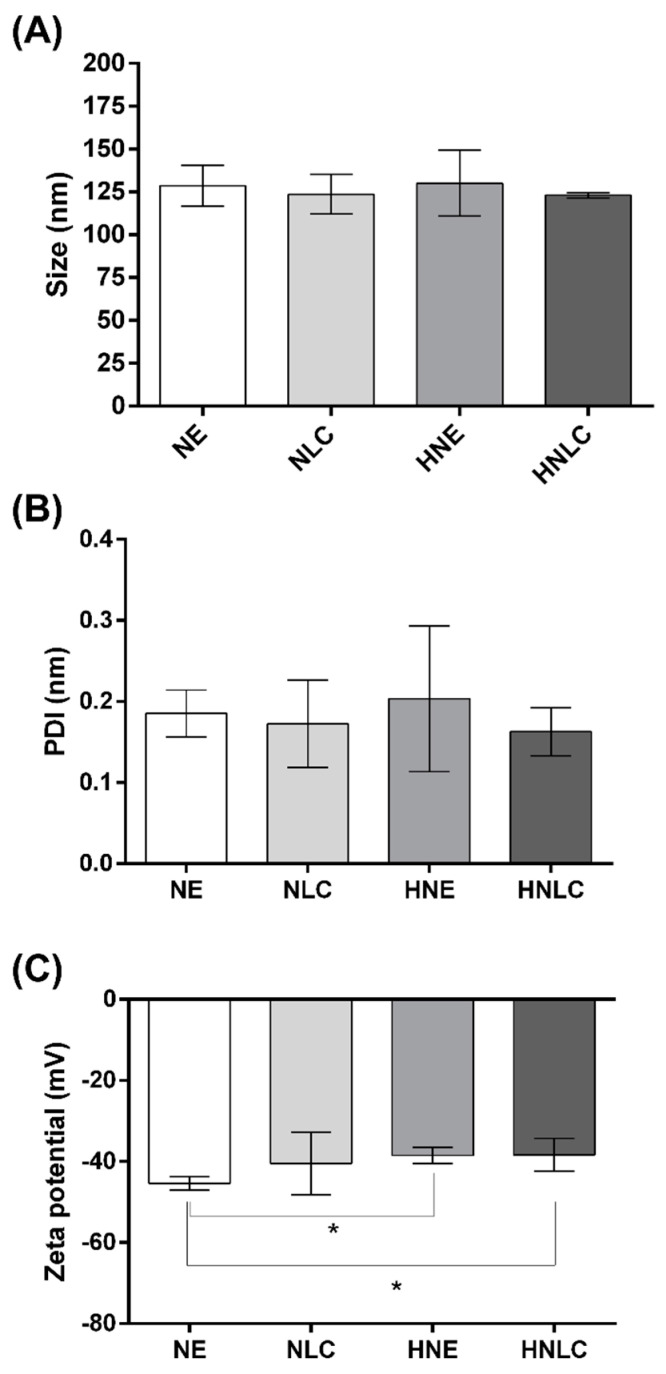
Results for particle size (nm) (**A**), polydispersity index (**B**), and zeta potential (mV) (**C**) for NE, NLC, HNE, and HNLC after preparation. Data are presented as mean ± SD (*n* = 3). NE: nanoemulsion containing EOPA; NLC: nanostructured lipid carrier containing EOPA; HNE: hydroxyethylcellulose hydrogel-thickened nanoemulsion containing EOPA; HNLC: hydroxyethylcellulose hydrogel-thickened nanostructured lipid carrier containing EOPA. Asterisks (*) represent statistically significant differences from NE, HNE, NLC, and HNLC by one-way ANOVA followed by Tukey’s post hoc test (*p* ≤ 0.05). No statistical difference in particle size and polydispersity index was observed between formulations (*p* ≥ 0.05).

NE and NLC loaded with EOPA were prepared by high-pressure homogenization due to advantages related to shorter production time and the absence of organic solvents [42,43,44]. Both *P. aduncum* loaded nanocarriers presented a milky and opaque appearance, woody odor, with droplet size in nanometric range (around 130 nm) (Figure 4). Then, the nanocarriers were thickened by hydroxyethylcellulose polymer in order to provide higher adherence and viscosity for topical administration [33,34,44]. The hydrogels developed maintained the original organoleptic characteristics of NE and NLC, with the added advantage of greater viscosity. HNE and HNLC presented droplet sizes of 130.16 ± 19.24 and 123.01 ± 1.65 nm, respectively. Nanometric size formulations have advantages related to broad absorption and increased permeability, besides minimizing adverse effects and toxic reactions [21,22].

In addition, a narrow polydispersity index below 0.3 for formulations was observed. NE and NLC presented PDI of about 0.18 ± 0.03 and 0.17 ± 0.05, respectively, while HNE and HNLC showed PDI of 0.20 ± 0.09 and 0.16 ± 0.03, respectively (Figure 4). In general terms, the narrow polydispersity index (<0.3) attributes monodispersion and uniformity to the nanoformulations, compatible with uniform colloidal system [27,28,32,45,46].

The results of zeta potential for NE, NLC, HNE, and HNLC were, respectively, −45.38 ± 1.65, −40.50 ± 7.69, −38.50 ± 2.03, and −38.34 ± 4.07 mV (Figure 4). The negative zeta potential of formulations may be attributed to the nonionic surfactant Span 80^®^ [47]. In addition, the zeta potential, with high values in module (>|30|), is known to provide electrostatic repulsion and, therefore, better nanocarrier stability [48].

**Figure 5:**
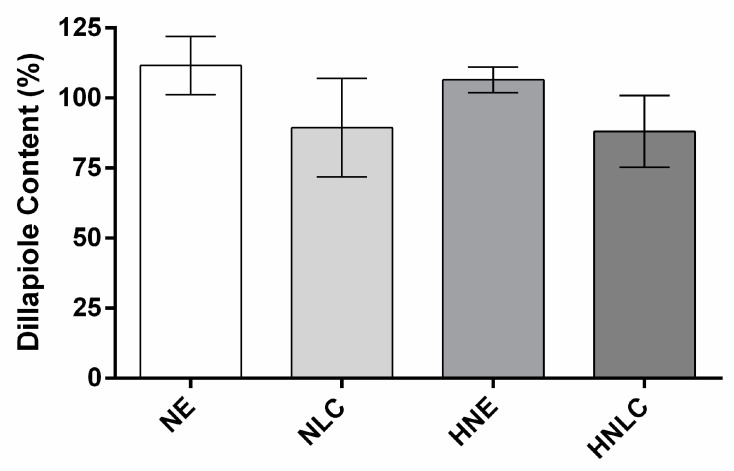
Dillapiole content (%) for NE, NLC, HNE, and HNLC after preparation relative to the initial value incorporated. Data are presented as mean ± SD (*n* = 3). NE: nanoemulsion containing EOPA; NLC: nanostructured lipid carrier containing EOPA; HNE: hydroxyethylcellulose hydrogel-thickened nanoemulsion containing EOPA; HNLC: hydroxyethylcellulose hydrogel-thickened nanostructured lipid carrier containing EOPA. No statistical difference in content (%) was observed between formulations (*p* ≥ 0.05).

The content of DIL from NE, NLC, and derived hydrogels was evaluated after preparation, and the results are presented in Figure 5. The average of the contents for the developed formulations was between 88.06% and 111.62%. The lower content for NLC and its derived hydrogel (89.42% and 88.06%, respectively), in comparison with NE and HNE (111.62% and 106.50%, respectively), although not statistically different, may be related to the production process that involved heating, which can promote some volatilization of EOPA during fabrication.

**Figure 6:**
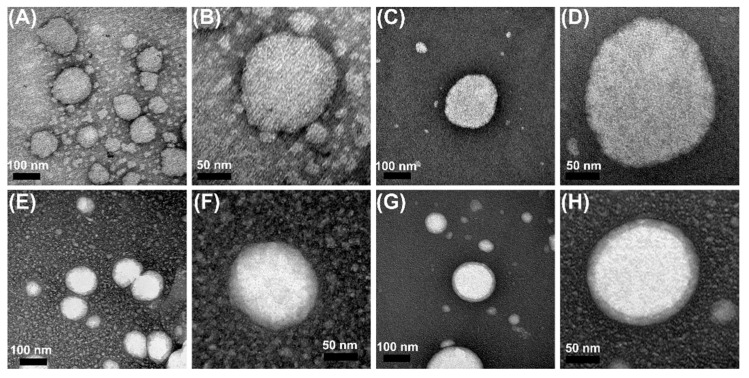
TEM micrographs at a magnification of 200 K (**A**,**C**,**E**,**G**) and 500 K (**B**,**D**,**F**,**H**) from NE (**A**,**B**), NLC (**C**,**D**), HNE (**E**,**F**), and HNLC (**G**,**H**). TEM: transmission electron microscopy; NE: nanoemulsion containing EOPA; NLC: nanostructured lipid carrier containing EOPA; HNE: hydrogel-thickened nanoemulsion containing EOPA; HNLC: hydrogel-thickened nanostructured lipid carrier containing EOPA.

The size and morphology of NE, NLC, HNE, and HNLC containing EOPA were investigated by transmission electron microscopy, and the images are compiled in Figure 6. The micrographs revealed particle sizes around 130 nm, indicating spherical, homogeneous, and smooth structures. For HNE and HNLC, a cloudy-like surface was also noted around the nanostructure, which may be related to the swelling of the polymer in the continuous phase of the nanosystems [44,49,50].

### 3.2. Rheological Behavior of Hydrogels Containing EOPA

**Figure 7:**
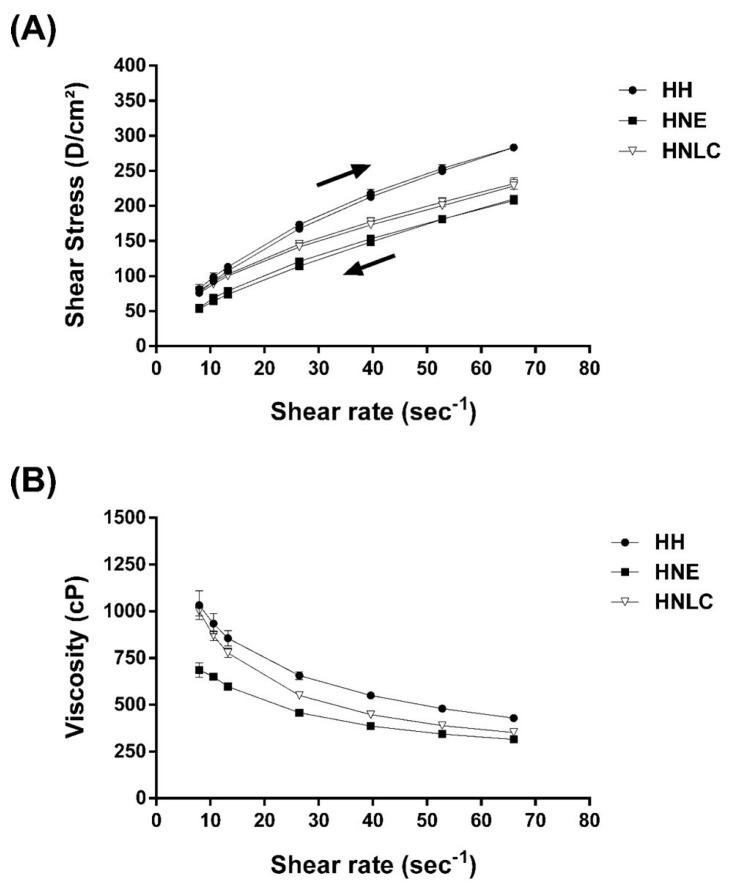
Rheological profile containing upcurves and downcurves (**A**) and viscosity profile (**B**) of blank hydroxyethylcellulose hydrogel without nanoemulsion or nanostructured lipid carrier (HH), hydroxyethylcellulose hydrogel-thickened nanoemulsion containing EOPA (HNE), and hydroxyethylcellulose hydrogel-thickened nanostructured lipid carrier containing EOPA (HNLC). Up arrow means upcurves, while down arrow means downcurves (Figure 7A).The results are presented as mean ± SD (*n* = 3).

The hydroxyethylcellulose hydrogel-thickened nanoemulsion and the hydroxyethylcellulose hydrogel-thickened nanostructured lipid carrier containing EOPA were characterized by their rheological profile, and results are shown in Figure 7 and Table 2.

As presented in the rheograms, the thickening of the nanoemulsion and nanostructured lipid carrier containing EOPA with the cellulose-derived polymer decreased the viscosity of the hydrogel when compared with hydroxyethylcellulose hydrogel (blank hydrogel, HH), which may be related to the higher viscosity and density relative to the water (Figure 7B). In addition, HNLC presented a higher viscosity in comparison with HNE, mainly related to the presence of a solid lipid in the oily core of HNLC (Figure 7B).

The rheograms for hydrogels presented in Figure 7 showed a non-Newtonian pseudoplastic behavior since viscosity is not constant, and it decreases with an increase in shear rate. To confirm the rheological behavior of formulations, different mathematical models (Bingham, Ostwald, Casson, and Herschel-Bulkley) were applied to the data in order to predict flow behavior (Table 2). The best-fit model selected for all formulations was the Ostwald flow model based on the coefficient of determination value (r^2^). From the Ostwald equation, the flow index value for HNE and HNLC was obtained from the slope of the equation, being, respectively, 0.62 and 0.503, which are lower than 1, confirming that both hydrogels have non-Newtonian flow with pseudoplastic behavior (Table 2) [51]. Additionally, a slight thixotropic behavior for HH, HNE, and HNLC was observed, as ascending and descending flow curves do not overlap, being related to a decrease in viscosity over time under a constant shear (viscosity time-dependent).

The non-Newtonian flow with the pseudoplastic behavior of the hydrogels containing EOPA is noteworthy in pharmaceutical products. The high viscosity and the thixotropic behavior of the formulations promote free flow when leaving the package and spreading on the skin, reduce the mobility of the dispersed phase and delay instability events [51].

### 3.3. Bioadhesion Measurements

**Figure 8:**
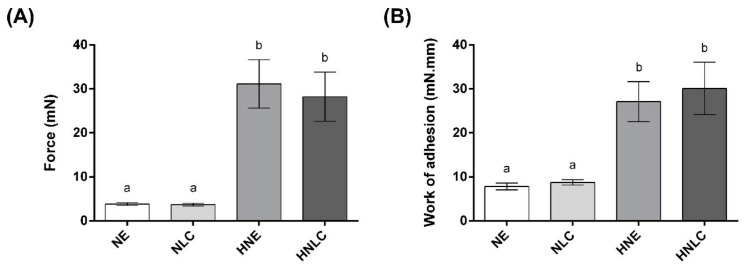
Bioadhesion measurements of force (**A**) and work of adhesion (**B**) for NE, NLC, HNE, and HNLC. Results are presented as mean ± SD (*n* = 6). Different letters mean statistical difference (*p* ≤ 0.05), while the same letters indicate no statistical difference (*p* ≥ 0.05). NE: nanoemulsion containing EOPA; NLC: nanostructured lipid carrier containing EOPA; HNE: hydroxyethylcellulose hydrogel-thickened nanoemulsion containing EOPA; HNLC: hydroxyethylcellulose hydrogel-thickened nanostructured lipid carrier containing EOPA. Different letters mean statistical difference (*p* ≤ 0.05), while the same letters indicate no statistical difference (*p* ≥ 0.05).

For this assay, porcine ear skin was used as biological tissue. The bioadhesion studies for NE, NLC, HNE, and HNLC are presented as a force of detachment and work of adhesion, as compiled in Figure 8. The work of adhesion (mN·mm), a direct measure of bioadhesion in tissues, was calculated from the area under the curve obtained by the relationship between the force of detachment (mN) versus the debonding distance (mm) [37].

The results demonstrated the same profile of force of detachment and work of adhesion for all the formulations tested (Figure 8). The hydrogels-thickened nanosystems proved to be more bioadhesive since work of adhesion and force of detachment were, respectively, about 8× and 3× higher for HNE and HNLC in comparison with NE and NLC, showing significant differences (*p* ≤ 0.05). In terms of aspect, NE and NLC are whitish liquid solutions with very low viscosity, while HNE and HNLC consist of 3D polymeric structures with higher viscosity and semisolid characteristics. The higher viscosity of the hydrogels is attributed to polymer swelling in the continuous phase of the nanoemulsion and nanostructured lipid carrier, which led to a greater force of detachment from the porcine skin [28,32]. Thus, the hydroxyethylcellulose polymeric network in hydrogels-thickened nanosystems is related to HNE and HNLC higher bioadhesiveness in comparison with NE and NLC, influencing mainly the residence time of the hydrogels in the porcine ear skin.

The bioadhesive characteristic is an interesting property for nanosystems, as the residence time can be prolonged, leading to a reduction in dose and frequency of application, and can also impact better patient compliance [52,53].

### 3.4. Dillapiole Release from the Formulations

**Figure 9:**
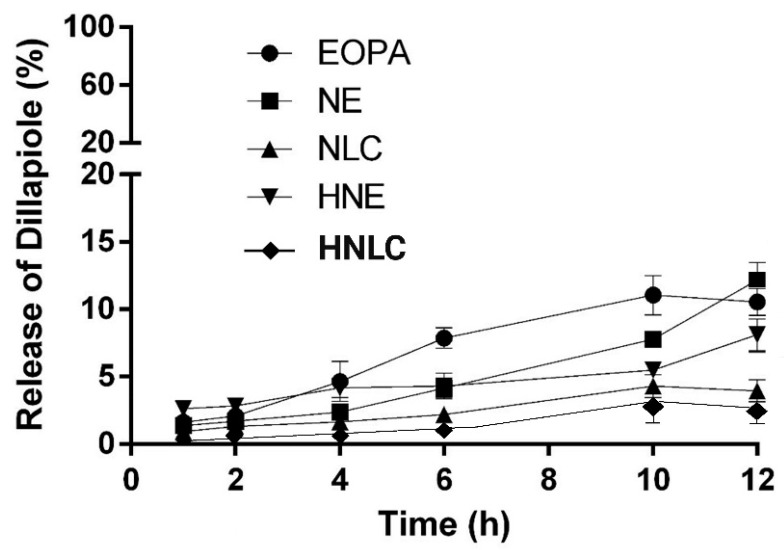
Release profile of DIL from EOPA, nanoemulsion containing EOPA (NE), nanostructured lipid carrier containing EOPA (NLC), hydrogel-thickened nanoemulsion containing EOPA (HNE), and hydrogel-thickened nanostructured lipid carrier containing EOPA (HNLC) (*n* = 3).

The release profiles of EOPA and nanosystems were evaluated in Franz-type diffusion cells with synthetic cellulose ester membranes, as shown in Figure 9.

During 12 h, aliquots of RF were collected and analyzed by HS-GC-FID. The total amount of DIL released from EOPA and formulations were estimated in percentage, and the results were 10.53 ± 0.45, 12.17 ± 0.50, 3.96 ± 0.81, 8.13 ± 0.47, and 2.77 ± 0.62 for EOPA, NE, NLC, HNE, and HNLC, respectively. The release of DIL from EOPA was faster in comparison with the formulations, especially at the first 6 h, achieving a plateau after 10 h (Figure 9). NE was the tested formulation that presented better results in terms of total DIL release. It was previously reported that nanoencapsulation can favor the delivery of active compounds due to nanometric size and high interfacial area [54].

Additionally, DIL from NE, NLC, HNE, and HNLC showed a controlled and constant release where the nanocarrier seems to provide a biphasic release pattern characterized by an initial burst (faster at the first 2 h), followed by a comparatively slower and continuous release (Figure 9). The release of DIL from NE was higher than NLC by about 3×, which may be related to the solid core of NLC, requiring a longer time for DIL to leave the oily core and diffuse through the membrane. Üner and coauthors reported differences between nanoemulsion and nanostructured lipid carriers on diffusion rates of celecoxib, attributing this effect to the mobility and malleability of droplets of nanoemulsion [55]. In addition, a low release was noted for HNLC and HNE in comparison with NE, almost 1.5×, mainly associated with the increased viscosity of the hydrogel [56]. It is noteworthy that the results should be interpreted with caution and do not necessarily reflect in vivo behavior. The membranes used have a pore size that is capable of separating the free compound from the nanoencapsulated one. In this sense, the compound has to partition out of the nanosystem to the external aqueous phase and then is able to diffuse through the membrane. However, this generates a low concentration of the compound in the aqueous phase, which is responsible for the low concentration gradient between the two sides of the membrane, limiting the compound release. Thus, the study is valid to compare formulations, in this case, to compare DIL release from EOPA (which is not associated with a nanosystem) and different formulations (nanosystems and nanosystems thickened in hydrogels).

### 3.5. In Vitro Permeation/Retention Studies

**Figure 10:**
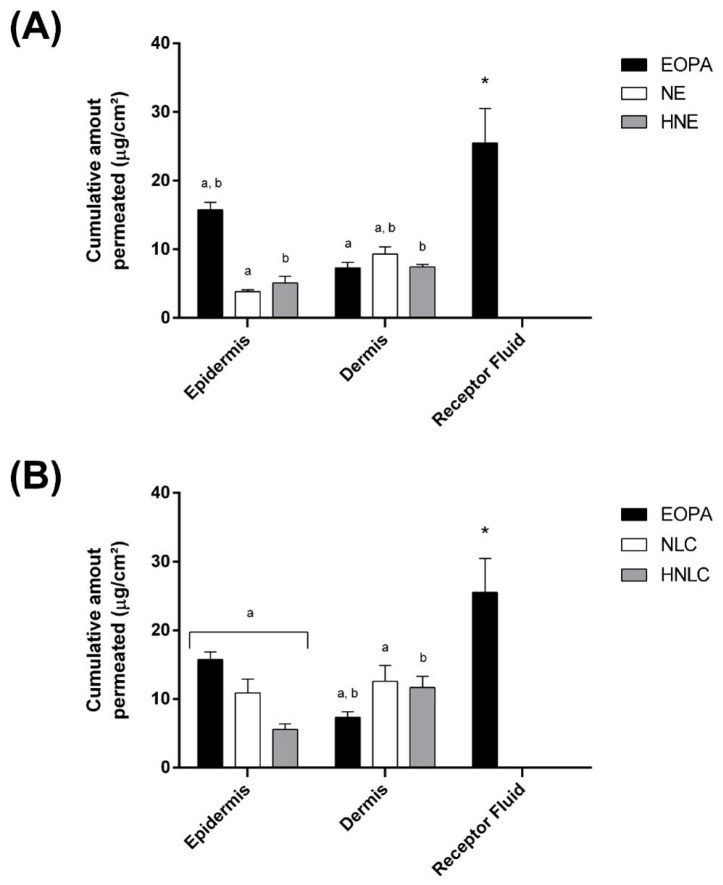
Cumulative amount permeated of dillapiole (**A**), nanoemulsion (**B**) containing EOPA (NE), nanostructured lipid carrier containing EOPA (NLC), hydrogel-thickened nanoemulsion containing EOPA (HNE), and hydrogel-thickened nanostructured lipid carrier containing EOPA (HNLC). After 8 h, DIL was not found in the stratum corneum for all tested substances. Results are presented as mean ± SD (*n* = 5). Same letters mean statistically significant difference by one-way ANOVA followed by Tukey’s post hoc test (*p* ≤ 0.05). Asterisks (*) represent statistically significant differences from NE, HNE, NLC, and HNLC by one-way ANOVA followed by Tukey’s post hoc test (*p* ≤ 0.05).

The in vitro permeation/retention studies for DIL from EOPA, NE, NLC, HNE, and HNLC were performed with Franz diffusion cell apparatus. Figure 10 shows the results of DIL permeation in the epidermis, dermis, and receptor fluid after the assay with porcine skin.

After 8 h, DIL was not found in the stratum corneum for all tested samples. For pure EOPA, the cumulative amount permeated of DIL was in order receptor fluid >> epidermis >> dermis. This result evidences the high capacity of DIL to penetrate deep tissue. Essential oils and their active compounds are able to overcome the cutaneous barrier by affecting the intercellular packing of the stratum corneum lipids, which could be an explanation for DIL permeation [57].

Furthermore, for NE, NLC, HNE, and HNLC, the cumulative amount permeated of DIL was in order dermis >> epidermis (Figure 10). As can be seen, for all formulations containing nanocarriers, even the ones thickened with hydroxyethylcellulose, the layer that most retained DIL was the dermis, attributing this effect to nanocarriers' ability to solubilize both hydrophilic and lipophilic substances, small size and large surface area, promoting the penetration to deeper layers [58]. This is desirable since there are several cell types responsible for inflammation and healing processes in the dermis [59]. Additionally, it was observed lower retention of DIL in the dermis from HNE and HNLC, in comparison with NE and NLC (Figure 10). Hydrogels-thickened nanocarriers may interfere with the delivery of active compounds due to high viscosity, causing active compounds to leave the polymeric network and then penetrate the skin [56].

Only for pure EOPA, dillapiole was found in the receptor fluid (Figure 10). The absorption of DIL from pure essential oil may impact toxicity and provoke unwanted systemic effects. As noted, for NE, NLC, HNE, and HNLC, DIL was not assayed in the receptor fluid, showing the ability of nanocarriers to decrease systemic absorption. Nanoformulations offer noteworthy advantages related to easier application, reduced systemic absorption, and enhanced selectivity to diseased skin in comparison to traditional treatments [60].

The results above are very promising when the intent is to apply formulations containing nanocarriers for the treatment of topical inflammatory processes.

### 3.6. Safety Profile by HET-CAM

The hen’s chorioallantoic membrane test (HET-CAM) is a simple, cheap, and in vitro alternative model to the use of animals able to evaluate the irritant potential of substances and formulations, including hydrogels, providing preliminary screening results of vascular events and safety assessment before in vivo study [39,40,61,62].

**Figure 11:**
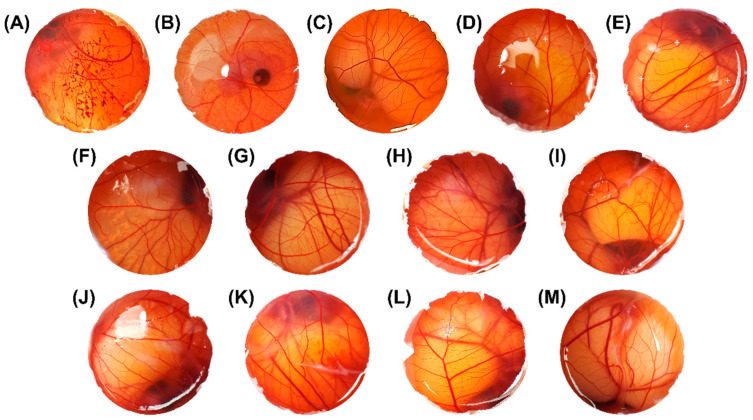
Sequence of photographs illustrating the effects of different substances applied on the chorioallantoic membrane over a 5 min period. (**A**) NaOH 0.1 M; (**B**) sodium lauryl sulfate 1% *w/v*; (**C**) NaCl 0.9% *w/v*; (**D**) olive oil; (**E**) essential oil of EOPA: olive oil (1:20 *v/v*); (**F**) blank nanoemulsion; (**G**) nanoemulsion containing EOPA; (**H**) blank nanostructured lipid carrier; (**I**) nanostructured lipid carrier containing EOPA; (**J**) blank hydrogel-thickened nanoemulsion; (**K**) hydrogel-thickened nanoemulsion containing EOPA; (**L**) blank hydrogel-thickened nanostructured lipid carrier; (**M**) hydrogel-thickened nanostructured lipid carrier containing EOPA. The marked (+) areas mean the exact position of the applied substance on the chorioallantoic membrane (CAM), while the unmarked areas refer to the entire CAM area.

Figure 11 and Table 3 present the irritation results in the CAM (*n* = 5). Sodium lauryl sulfate 1% *w/v* and sodium hydroxide (NaOH) 0.1 M were applied as a positive control for vasoconstriction, hemorrhage, and coagulation, respectively. Irritation scores for sodium lauryl sulfate and NaOH were, respectively, 11.14 and 13.51, both being extremely irritating. Additionally, as a negative control (non-irritating), sodium chloride solution (NaCl 0.9% *w/v*) was used, which an irritation score was 0, as expected, being non-irritant for the CAM. To solubilize EOPA at the same proportion of the nanosystems, olive oil was applied since it is considered a non-irritant control and presented an irritation score of 0, as expected [63]. The blank formulations without the essential oil (B-NE, B-NLC, HB-NE, and HB-NLC) had an irritation score of 0, being considered non-irritating, confirming that the surfactants and polymer did not influence the toxicity in the formulations. Meanwhile, EOPA solubilized in olive oil in the proportion of 1:20 showed a slight irritation score of 4.38. In addition, NLC containing DIL and its derived hydrogel (HNLC) showed a slight irritation score of 4.68 and 4.82, respectively, while the same pattern was observed for the NE and derived hydrogel (HNE), 4.68 and 4.82, respectively. These results suggest that the formulations containing EOPA presented a low irritation score, which may be related to its compounds, including DIL. In previous studies, EOPA was considered genotoxically safe [7]. In contrast, the toxicity of EOPA against *Rhipicephalus microplus* larvae has been described [64]. It has also been reported that the EOPA is able to increase the osmotic fragility curve of red blood cells [65]. These findings indicate that the essential oil has indeed a correlation with the irritant potential found in the HET-CAM assay; however, the literature does not present a clear explanation about the probable irritation cause for EOPA. It is important to highlight that toxicity and irritability of substances are dependent on various factors, even composition, and concentration [65]. In this sense, as a suggestion for low-irritant substances, Oliveira and coauthors recommended a cut-off point of 4.9, indicating a low irritant potential for formulations below this value [66]. Thereby, as IS scores for all formulations containing EOPA (NE, NLC, HNE, and HNLC) were lower than 4.9, the developed nanosystems and derived hydrogels were considered safe to be applied topically.

## 4. Conclusions

The formulations developed presented nanometric particle size, narrow polydispersity index, negative zeta potential, and adequate content of dillapiole relative to the initial value added. Furthermore, the hydrogels-thickened NE and NLC exhibited pseudoplastic behavior with bioadhesive properties. In addition, all the nanoformulations presented a controlled release profile, the ability to deliver dillapiole to the dermis, and low irritant potential. The promising results regarding the permeation to deeper layers of dillapiole from EOPA loaded in nanocarriers and derived hydrogels proved to be an interesting strategy for the treatment of inflammatory processes.

## Figures and Tables

**Table 1:** Composition of formulations containing or not EOPA (%, *w/w*).

Constituents	B-NE	B-NLC	NE	NLC	HB	HB-NE	HB-NLC	HNE	HNLC
EOPA	-	-	5	5	-	-	-	5	5
MCT	10	5	5	-	-	10	5	5	-
Span 80^®^	2.5	2.5	2.5	2.5	-	2.5	2.5	2.5	2.5
Tween 20^®^	2.5	2.5	2.5	2.5	-	2.5	2.5	2.5	2.5
Cupuaçu butter	-	5	-	5	-	-	5	-	5
Hydroxyethylcellulose	-	-	-	-	1	1	1	1	1
Water q.s.	100	100	100	100	100	100	100	100	100

**Table 2:** Equations and coefficients of determination (r^2^) for rheological flow models based on shear rate and shear stress curves from hydrogels.

Formulation	Bingham Model		Ostwald Model		Casson Model		Herschel-Bulkley Model	
	Equation	r^2^	Equation	r^2^	Equation	r^2^	Equation	r^2^
HH	y = 3.4917x + 66.59	0.98	y = 0.5886x + 1.3917	0.9988	y = 1.4749x + 5.2063	0.9919	y = 1.3241x − 0.0281	0.9667
HNE	y = 2.6076x + 42.919	0.9868	y = 0.62x + 1.1939	0.9983	y = 1.3043x + 4.0222	0.9941	y = 1.258x − 0.0475	0.9791
HNLC	y = 2.6088x + 67.061	0.9868	y = 0.503x + 1.4476	0.9999	y = 1.1831x + 5.7667	0.9965	y = 1.3193x − 0.1526	0.9718

HH: blank hydroxyethylcellulose hydrogel without nanoemulsion or nanostructured lipid carrier; HNE: hydroxyethylcellulose hydrogel-thickened nanoemulsion containing EOPA; HNLC: hydroxyethylcellulose hydrogel-thickened nanostructured lipid carrier containing EOPA; r^2^: coefficient of determination.

**Table 3:** Classification of cumulative scores in the chorioallantoic membrane test (HET-CAM) (*n* = 5).

	IS (RSD%)	Results
NaCl 0.9% m/v	0 (0)	Non-irritant
Lauryl sodium sulfate 1% m/v	11.14 (0.62)	Extremely irritant
NaOH 0.1 M	13.51 (1.15)	Extremely irritant
Olive oil	0 (0)	Non-irritant
EOPA: Olive oil (1:20)	4.38 (3.61)	Slightly irritant
B-NE	0 (0)	Non-irritant
NE	4.08 (9.88)	Slightly irritant
HB-NE	0 (0)	Non-irritant
HNE	4.81 (0.38)	Slightly irritant
B-NLC	0 (0)	Non-irritant
NLC	4.68 (6.77)	Slightly irritant
HB-NLC	0 (0)	Non-irritant
HNLC	4.82 (0.29)	Slightly irritant

IS: irritation score; RSD%: relative standard deviation in percentage; B-NE: blank nanoemulsion; B-NLC: blank nanostructured lipid carrier; NE: nanoemulsion containing EOPA; NLC: nanostructured lipid carrier containing EOPA; HB: hydroxyethylcellulose hydrogel; HB-NE: blank hydrogel-thickened nanoemulsion; HB-NLC: blank hydrogel-thickened nanostructured lipid carrier; HNE: hydrogel-thickened nanoemulsion containing EOPA; HNLC: hydrogel-thickened nanostructured lipid carrier containing EOPA.

## Supplementary Materials

The following supporting information can be downloaded at: https://www.mdpi.com/article/10.3390/pharmaceutics14112525/s1, Figure S1: *P. aduncum* essential oil chromatogram performed by gas chromatography coupled to mass spectrometry. The compounds are (1) β-caryophyllene 10.65%, (2) bicyclogermacrene 2.18%, (3) n-pentadecane 7.12% and (4) dillapiole 80.05%. RT: Retention Time; RI calc.: Retention Index calculated; RI lit.: Retention Index from literature [36]; KI calc.: Kovats Index calculated; KI lit.: Kovats Index from literature [36].
