# Supplementary material for: Piper aduncum Essential Oil Rich in Dillapiole: Development of Hydrogel-Thickened Nanoemulsion and Nanostructured Lipid Carrier Intended for Skin Delivery
Source: Pharmaceutics. 2022 Nov 19;14(11):2525. doi: 10.3390/pharmaceutics14112525 (PMC9696712; doi:10.3390/pharmaceutics14112525)
Supplement: Supplementary file 1 [file pharmaceutics-14-02525-s001.zip › pharmaceutics-2006010-supplementary.pdf]

SUPPLEMENTARY MATERIAL

Piper aduncum essential oil rich in dillapiole: development of hydrogel-thickened nanoemulsion and nanostructured lipid carrier intended for skin delivery

Simone Braga Carneiro[1†], Tainá Kreutz[2†], Renata Pereira Limberger[2], Helder Ferreira Teixeira[2], Valdir Florêncio da Veiga Júnior[3], Letícia Scherer Koester[2*]

Affiliation

1 Programa de Pós-Graduação em Inovação Farmacêutica, Faculdade de Ciências Farmacêuticas, Universidade Federal do Amazonas, Av. Gal. Rodrigo Octávio, Coroado I, 1200, Zip code 69067-005, Manaus, Amazonas, Brazil

2 Programa de Pós-Graduação em Ciências Farmacêuticas, Faculdade de Farmácia, Universidade Federal do Rio Grande do Sul, Av. Ipiranga, Santana, 2752, Zip code 90610-000, Porto Alegre, Rio Grande do Sul, Brazil

3 Programa de Pós-Graduação em Quí mica,Instituto Militar de Engenharia, Praça General Tibúrcio, Urca, 80, Zip code 22290-270, Rio de Janeiro, Rio de Janeiro, Brazil

* Correspondence: leticia.koester@ufrgs.br.

† Both authors contributed equally to this manuscript.

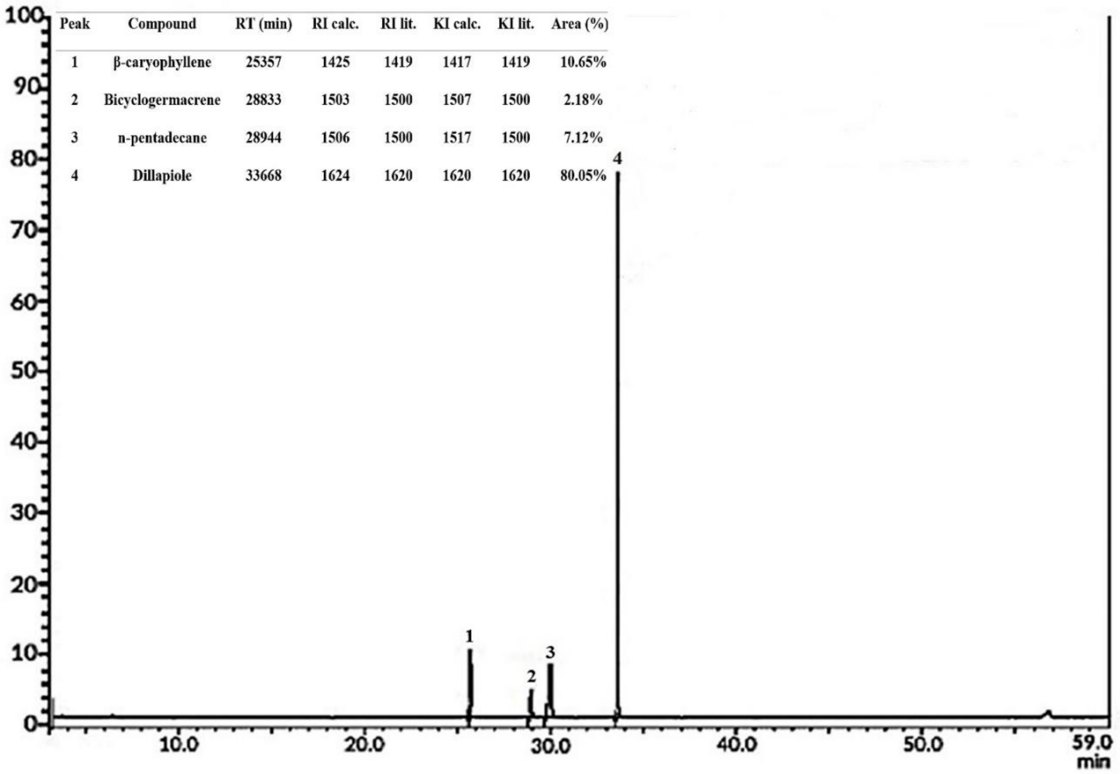

Peak	Compound	RT (min)	RI calc.	RI lit.	KI calc.	KI lit.	Area (%)
1	β-caryophyllene	25357	1425	1419	1417	1419	10.65%
2	Bicyclogermacrene	28833	1503	1500	1507	1500	2.18%
3	n-pentadecane	28944	1506	1500	1517	1500	7.12%
4	Dillapiole	33668	1624	1620	1620	1620	80.05%

Figure S1. P. *aduncum* essential oil chromatogram performed by gas chromatography coupled to mass spectrometry. The compounds are (1) β-caryophyllene 10.65%, (2) bicyclogermacrene 2.18%, (3) n-pentadecane 7.12% and (4) dillapiole 80.05%. RT: Retention Time; RI calc.: Retention Index calculated; RI lit.: Retention Index from literature [36]; KI calc.: Kovats Index calculated; KI lit.: Kovats Index from literature [36].
